# Electrical and Self-Sensing Properties of Alkali-Activated Slag Composite with Graphite Filler

**DOI:** 10.3390/ma12101616

**Published:** 2019-05-16

**Authors:** Pavel Rovnaník, Ivo Kusák, Patrik Bayer, Pavel Schmid, Lukáš Fiala

**Affiliations:** 1 Faculty of Civil Engineering, Brno University of Technology, Veveří 95, 602 00 Brno, Czech Republic; kusak.i@fce.vutbr.cz (I.K.); bayer.p@fce.vutbr.cz (P.B.); schmid.p@fce.vutbr.cz (P.S.); 2 Faculty of Civil Engineering, Czech Technical University, Thákurova 7, 166 29 Prague, Czech Republic; fialal@fsv.cvut.cz

**Keywords:** alkali-activated slag, graphite, electrical resistivity, self-sensing, compressive strength, microstructure

## Abstract

The electrical properties of concrete are gaining their importance for the application in building construction. In this study, graphite powder was added to alkali-activated slag mortar as an electrically conductive filler in order to enhance the mortar’s conductive properties. The amount of graphite ranged from 1% to 30% of the slag mass. The effect of the graphite powder on the resistivity, capacitance, mechanical properties, and microstructure of the composite was investigated. Selected mixtures were then used for the testing of self-sensing properties under compressive loading. The results show that the addition of an amount of graphite equal to up to 10% of the slag mass improved the electrical properties of the alkali-activated slag. Higher amounts of filler did not provide any further improvement in electrical properties at lower AC frequencies but caused a strong deterioration in mechanical properties. The best self-sensing properties were achieved for the mixture with 10 wt% of graphite, but only at low compressive stresses of up to 6 MPa.

## 1. Introduction

Electrically conductive composite materials applicable in the building industry have been very extensively studied during the last three decades [1,2,3]. The studies have been generally concerned with the behaviour of these materials in various electric fields (DC, AC with various frequencies) and determine a variety of material properties that depend on changes in electrical resistivity. Therefore, it is necessary to design advanced cement-based composites and to conduct electrical resistivity measurements and analysis before practical applications can be considered.

With regard to the electrical measurement of cement-based materials, one has to take into account that concrete is a very good electrical insulator: The electrical resistivity is up to 10^9^ Ωm for oven-dried concrete (105 °C) [4,5]. The addition of electrically conductive substances to cement composites in order to achieve an increase in their flexural strength, such as steel fibres or carbon fibres, leads to a decrease in electrical resistivity [6]. In the case that the primary aim is not the improvement of strength properties, but a decrease in electrical resistivity, one can use a variety of conductive components: Steel slag, graphite powder, carbon black, carbon nanofibers, carbon nanotubes, or nickel powder [7,8].

The decrease in the electrical resistivity of concrete caused by electrically conductive fillings opens up new application possibilities that include the development of self-sensing [1], self-heating [9] and snow-melting composites [10,11,12], security composites that eliminate electric signal throughput [13,14], or composites that can be used to determine the weight of moving objects [7,15]. The self-sensing properties of concrete can be measured under uni- or biaxial compression, tension or flexure. Under monotonous compression, the electrical resistivity at first decreases upon loading as the particles of functional fillers are pushed closer to each other, thus improving the conductive network. Then, the resistivity stays balanced due to the simultaneous initiation and compression of fresh cracks, which lead to the destruction and reconstruction of the conductive network. Finally, the resistivity increases abruptly when large cracks propagate resulting in a total breakdown of conductive pathways [8].

Graphite is a carbon polymorph with a layered, planar structure. Individual layers of graphite are called graphene; within them the carbon atoms are arranged in a hexagonal lattice. These layers exhibit good conductivity along the planes due to the free mobility of electrons located in delocalized π-molecular orbitals. On the other hand, transversal conductivity is very low because the distance between graphene planes is 335.4 pm and the bond between them is only a weak van der Waals interaction. However, if the graphite is pressed perpendicular to the planes, these get closer to each other, which enables the easier migration of electrons between layers and leads to the enhancement of tunnelling conduction [16]. Graphite has already been used as a conductive functional filler in the fabrication of asphalt concrete with sensing properties. It dramatically decreased the electrical resistivity of this type of material and endowed it with self-sensing ability [17,18]. Graphite has also been used to enhance the electrical conductivity and internal damage monitoring of concrete structures either as the only conductive admixture or in combination with carbon fibres [16,19,20]. Sun et al. [16] used nano graphite platelets as a conductive admixture and showed that the highest gauge factor, and hence sensing performance, is reached with 5 wt% of this type of graphite admixture. The piezo-resistive behaviour of this composite is highly reproducible and suggests it may be suitable for use in sensors for the health monitoring of concrete structures and the assessment of their physical state.

Despite the great attention paid to the investigation of cement-based materials with electrically conductive admixtures, only a little work has been devoted to alkali-activated aluminosilicate or geopolymer-based composites [21,22,23,24,25]. Alkali-activated slag (AAS) has been used from the early 1960s as an alternative binder to Portland cement. It is composed of ground granulated blast furnace slag and alkaline activator, which is usually hydroxide, carbonate or silicate. Since the binder utilizes the waste as a primary aluminosilicate source, its environmental impact and global warming potential are much lower than that of ordinary concrete [26,27]. Its properties such as high corrosion resistance [28,29,30,31,32] or enhanced resistance to high temperatures and fire [33,34,35,36,37] predetermine this material for use in special applications where superior properties are needed. Current trends in the development of smart structures place high demands on the application of new materials with enhanced electrical properties. This also brings the need for research of the electrical properties of alkali-activated materials and its composites with electrically conductive fillers.

This work presents a comprehensive investigation of the electrical and self-sensing properties of alkali-activated slag composite with graphite powder as a conductive filler. A wide range of graphite dosage (1%–30% of the slag mass) was used in this study in order to determine the percolation threshold concerning the electrical properties which is the crucial parameter in designing the conductive filler dosage for the self-sensing applications. While the resistivity well below the percolation threshold is needed for the self-monitoring of the material, the possible highest conductivity is required for the self-heating or snow-melting applications [38], and in order to eliminate the electromagnetic signal throughput even larger amounts of filler may be needed. The specific value of the resistivity also strongly depends on the moisture content [39]. The overall performance of this composite was also supported by microstructural investigation and the determination of mechanical properties. Although the graphite powder is rather expensive compared to the AAS binder and it is not very likely to use such large amount of filler in practice, the investigation brings an interesting overview of the complex influence of graphite on the properties of AAS composite that fills the knowledge gap in this field and can help with the design and application of these perspective materials in smart structures.

## 2. Experimental Part

### 2.1. Materials

The tested material was prepared via the alkali activation of granulated blast furnace slag. The slag was supplied by Kotouč (CZ) and its specific surface area was 383 m^2^ kg (Blaine). The average grain size of the slag particles obtained by laser granulometry was *d*_50_ = 15.5 µm and *d*_90_ = 38.3 µm. SUSIL MP 2.0 solid sodium water glass (Vodní sklo, Praha, Czech Republic) with an SiO_2_/Na_2_O ratio of 2 was used as an alkaline activator. The chemical composition of the slag is given in Table 1.

**Figure 1:**
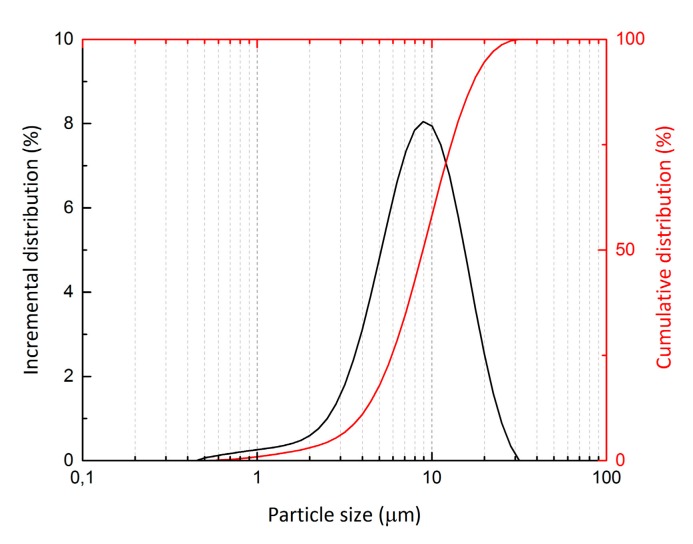
Particle size distribution of graphite powder obtained via laser granulometry.

Standard quartz sand with a maximum grain size of 2.5 mm was used as filler in order to prepare AAS mortars. Graphite powder (COND 96-8, Graphite Týn, Týn nad Vltavou, Czech Republic) was added to the AAS composite in order to increase electrical conductivity. Its particle size distribution determined by laser granulometry is presented in Figure 1 and the average grain size was *d*_50_ = 6.4 µm and *d*_90_ = 13.0 µm. The Brunauer–Emmett–Teller (BET) surface area [40] of the graphite determined by nitrogen absorption was 15.42 m^2^ g^−1^. Triton X-100 (Sigma-Aldrich, St. Louis, MO, USA) was used as a dispersing agent for the graphite powder. It was added to the graphite suspension in the form of a 0.5% solution. In order to avoid the formation of large pores due to air entrapped during mixing, a 1% solution of the siloxane-based air-detraining agent, Lukosan S (Lučební závody, Kolín, Czech Republic), was used.

### 2.2. Sample Preparation and Testing Procedures

The composition of the reference mixture was based on our previous experience due to its very good mechanical performance and user-friendly application [33,34,35]. First, a homogenous suspension of graphite powder was prepared with 0.5% aqueous solution of Triton X-100 as a dispersant. Sodium silicate activator was suspended and partially dissolved in water, and then mixed with prepared graphite suspension and stirred in a planetary mixer for 2 min. Then, slag and quartz aggregate were added and the mixture was stirred for about 5 min to prepare fresh mortar. Finally, defoamer was mixed into the mortar in order to prevent bubbles and additional water was added to adjust the designed workability of the mixture. This way of mixing was used following the assumption that addition of graphite in the beginning of the mixing process could improve the homogenous dispersion of the graphite particles. The amount of activator added was 20% of the slag mass, the aggregate/slag ratio was equal to 3 and water content varied with the amount of graphite to achieve approximately the same consistency measured by slump. The graphite content ranged from 1% to 30% of the slag mass; the composition of the mixtures is presented in Table 2.

**Figure 2:**
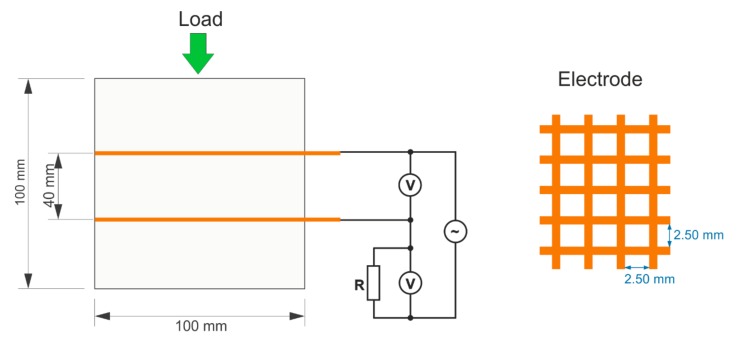
Experimental setup for the measurement of self-sensing properties under compression.

Two types of specimens were prepared—the prismatic specimens of the size 40 mm × 40 mm × 160 mm were used for the measurement of electrical properties and mechanical tests and 100 mm × 100 mm × 100 mm cubes with embedded electrodes were used for the investigation of self-sensing properties. The gauze electrodes were made of copper with a wire thickness of 1 mm and a mesh size of 2.5 mm. The size of the electrodes was 80 mm × 120 mm and the distance between them was 40 mm (Figure 2).

The hardened specimens were cured in a water bath at 20 °C for 28 days. The prismatic specimens for the testing of electrical properties were dried at 105 °C to a constant weight in order to avoid the influence of moisture. The cubes for the measurement of self-sensing properties were stored under ambient conditions for 6 weeks before testing in order to achieve moisture equilibrium.

Impedance spectroscopy in the range of 40 Hz to 1 MHz was used to analyse the electrical properties of the prepared samples. The measurements were made using an Agilent 33220A sinusoidal signal generator with an output voltage of 5.5 V and an Agilent 54645A dual-channel oscilloscope. The input values for the electrical capacity and the resistance of the oscilloscope were 13 pF and 1 MΩ, respectively. In order to perform impedance analysis, the prismatic specimens were placed between parallel brass electrodes (30 mm × 100 mm) so that the distance between the electrodes was 40 mm.

The complex impedance that was measured as a function of frequency can be written as:

(1) $$Z^{*}=R+iX_{c},$$

where *R* is electrical resistance and *X*_c_ is capacitive reactance. Since the electrical resistance depends on the size of the sample, the electrical and sensing behaviour of the materials is usually expressed in terms of the volume electrical resistivity:

(2) $$\rho =R\cdot \frac{S}{l},$$

where *S* is the surface area of the electrodes and *l* is the distance between them. The capacitance was calculated according to Equation (3), where *f* is the frequency of the AC source.

(3) $$C=\frac{1}{2\pi f\cdot X_{C}}.$$

A FORM+TEST Prüfsysteme hydraulic testing machine with a measuring range of 0–3000 N was used to measure the sensing properties on the cubic specimens that were loaded perpendicular to the plane of the electrodes (Figure 2). The repeated linear loading and releasing of the samples was performed with a loading rate of 400 N s^−1^ and in the range of 5–50 kN. In the final experiment, the cubes were tested under linear loading with a loading rate of 200 N s^−1^ up to failure.

For the measurement of the electrical resistance of the loaded samples, an Agilent 33220A sinusoidal signal generator and two Agilent 34410A multimeters (Agilent Technologies, Santa Clara, CA, USA) were used in the arrangement depicted in Figure 2. The sensing properties were measured in both a DC and an AC field. The frequencies of the AC field were 50 Hz and 1 kHz and the input voltage was 5 V. The current was calculated using Ohm’s law from the voltage measured on the reference resistance *R* = 6796 Ω. The electrical resistance of the measured sample was computed from the calculated electric current and the voltage that was measured separately on the test sample. The internal resistance of the voltmeter was taken into account for the calculation as well.

The specimens were also tested for their residual mechanical properties. Flexural strengths were determined on the prismatic specimens using a standard three-point-bending test and compressive strengths were measured on the far edge of each of the two residual pieces obtained from the flexural test [41].

Pore distribution was evaluated by means of mercury intrusion porosimetry (MIP) analysis, which was conducted on samples using a Micromeritics Poresizer 9310 porosimeter that can evaluate a theoretical pore diameter of 6 nm. Micrographs of the alkali-activated slag mortars were taken on a TESCAN MIRA3 XMU scanning electron microscope in secondary electron imaging mode. Secondary electron imaging was carried out on samples that were sputtered with gold. Samples for the MIP and SEM analyses were dried at 60 °C for 96 h prior to measurement in order to release free moisture.

## 3. Results

### 3.1. Mechanical Properties

**Figure 3:**
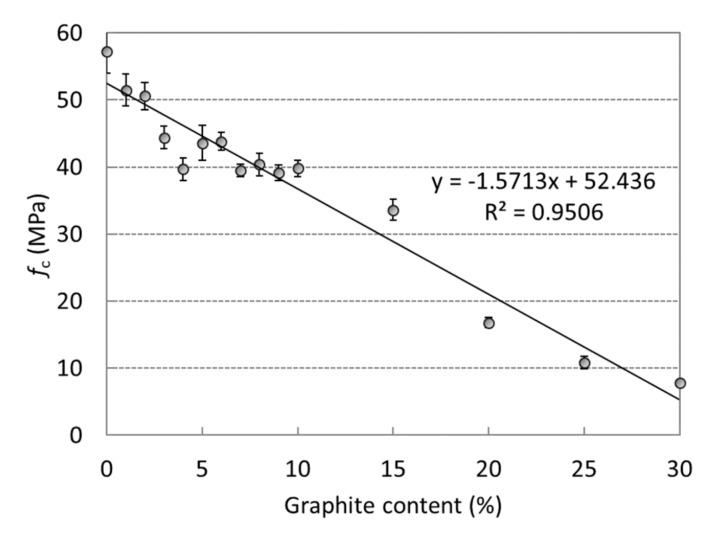
Compressive strengths of alkali-activated slag (AAS) composites with 0 wt%–30 wt% of graphite powder (standard deviations are presented as error bars).

**Figure 4:**
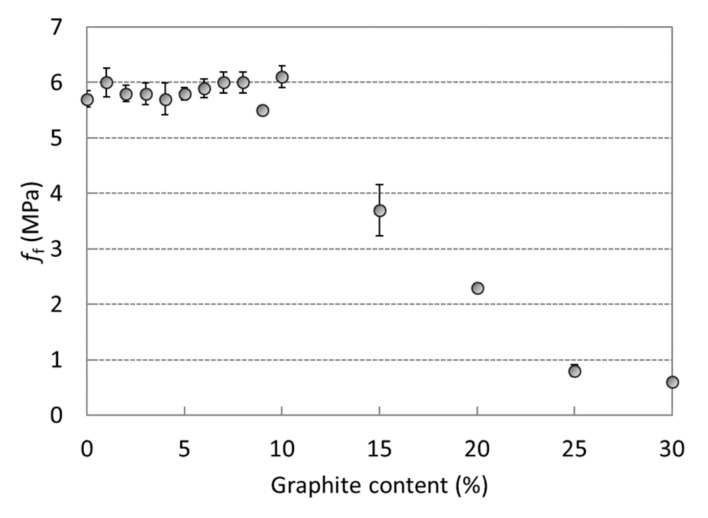
Flexural strengths of AAS composites with 0 wt%–30 wt% of graphite powder (standard deviations are presented as error bars).

Compressive strengths for the composites with different amounts of graphite filler are presented in Figure 3. The compressive strength of the reference mortar assigned as G0 was 57 MPa. Considerable strength deterioration that was observed after the addition of graphite is associated with the higher water-to-slag ratio and the rather weak bond between the graphite particles and the AAS matrix. The results show an almost linear decrease in strength, with the lowest value of 8 MPa achieved for mixture G30. It is only 14% of the reference strength. The deviation from linearity can be attributed to the differences in the amount of organic admixtures. The corresponding flexural strengths were not affected when the amount of graphite was below 10% of the slag mass and the average value was 5.8 MPa (Figure 4). However, with higher wt% of added graphite the flexural strength of the AAS composite linearly decreased to reach a minimum of 0.6 MPa for the mixture with the highest amount of graphite. This can be attributed to the weaker bond between the polar AAS matrix and the hydrophobic graphite particles, and also to the lamellar structure of graphite, which can act as a stress concentrator for crack propagation.

**Figure 5:**
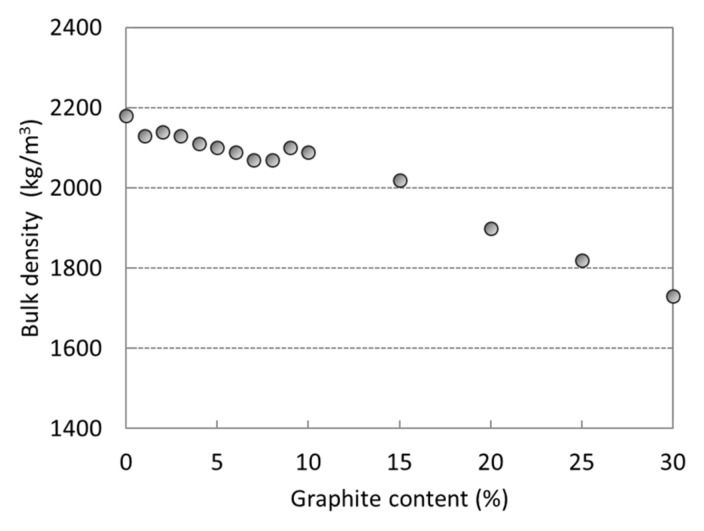
Bulk density of AAS composites with 0 wt%–30 wt% of graphite powder.

A similar trend as in the case of flexural strength was also observed for the bulk density measurements (Figure 5). After the addition of graphite in amounts of up to 10% of the slag mass, the bulk density decreased only by 5%. Due to the much lower density of graphite compared to the slag, higher amounts of graphite caused a significant (20% maximum) decrease in the bulk density of the AAS composite.

### 3.2. Electrical Properties

**Figure 6:**
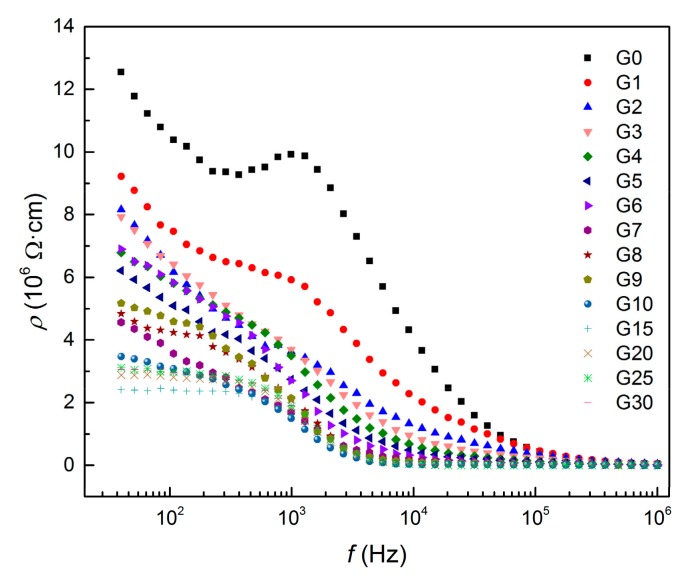
Electrical resistivity of AAS composites with 0 wt%–30 wt% of graphite powder as a function of frequency.

**Figure 7:**
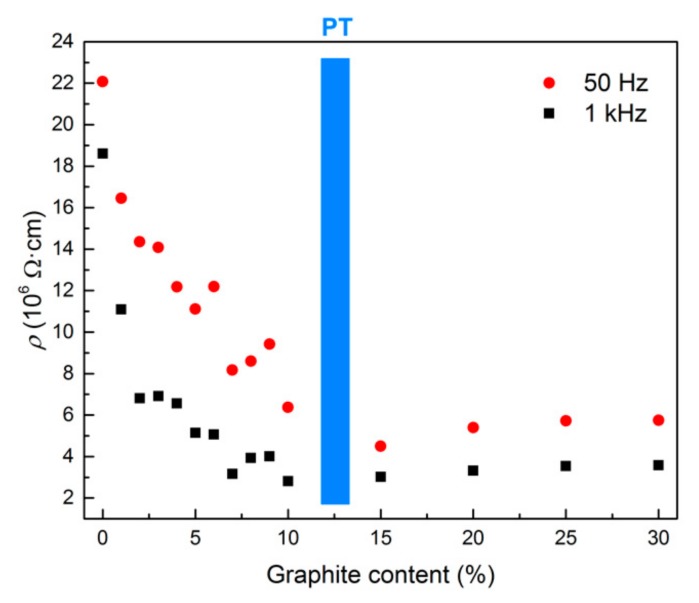
Change in the electrical resistivity of AAS composites with respect to graphite content at 50 Hz and 1 kHz (PT = percolation threshold).

Electrical properties were measured in AC mode in two different frequency ranges. Figure 6 illustrates the resistivity with increasing amounts of graphite powder in the frequency range of 40 Hz–1 MHz. The resistivity decreased as the amount of graphite rose and with the frequency of the AC source. There is a significant peak located around 1 kHz in the curve measured for the reference mixture and this feature can also be observed as a small plateau for the mixture with 1 wt% of graphite. With higher amounts of graphite, only a gradual decrease in the resistivity with no specific features was observed. The resistivity of the composites with a very high content of graphite powder (over 10 wt%) reached its minimum values at relatively lower frequencies below 10 kHz; however, at frequencies over 100 kHz, the differences in resistivity of all tested composites diminished. In order to analyse the effect of graphite content on the electrical resistance of the samples, two frequencies (50 Hz and 1 kHz) were chosen. It is obvious that the trend is independent of the frequency used. Figure 7 shows that even a small amount of added graphite caused an essential decrease in electrical resistivity. The resistivity dropped as the amount of conductive filler increased, and reached its minimum between 10 wt%–15 wt% of graphite. It can be assumed that such an amount of graphite is high enough to arrange the contacts between neighbouring particles, and therefore contact conductance becomes dominant. For that reason, adding more graphite has a negligible effect on the resistivity of AAS composite.

**Figure 8:**
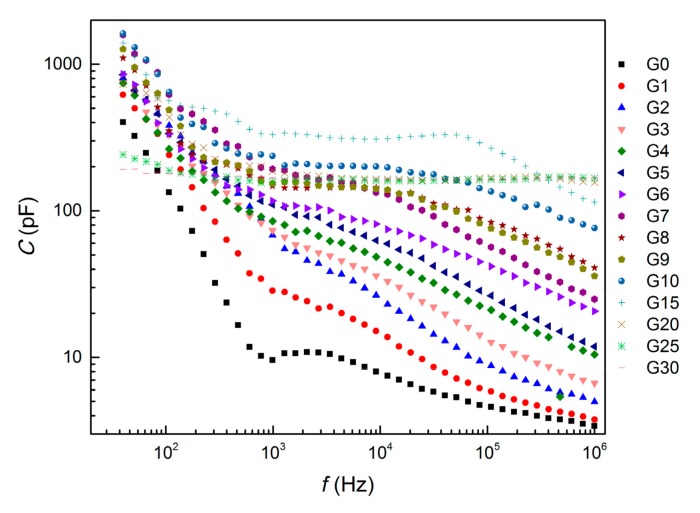
Electrical capacitance of AAS composites with 0 wt%–30 wt% of graphite powder as a function of frequency.

**Figure 9:**
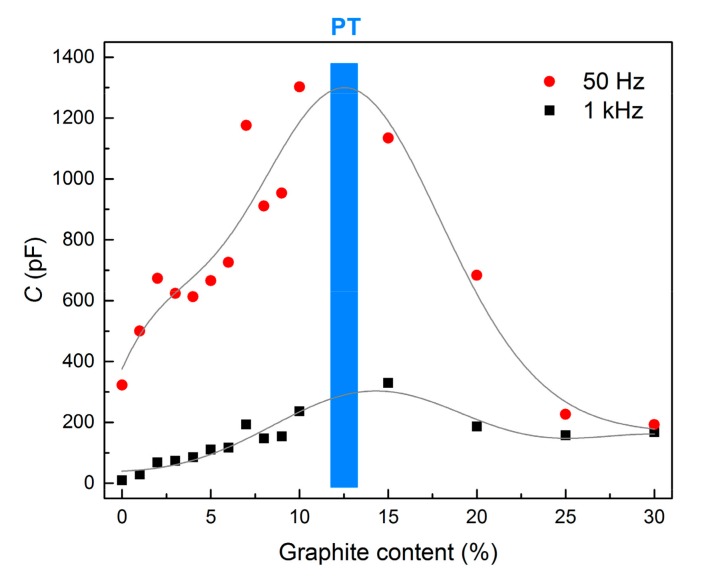
Change in the electrical capacitance of AAS composites with respect to graphite content at 50 Hz and 1 kHz (PT = percolation threshold).

The electrical capacitance of the samples as a function of AC frequency is presented in Figure 8. The capacitance firstly rose with increasing amounts of graphite admixture because graphite particles are able to absorb the electrical charge, either by the formation of dipole elements or as a free charge carrier. Since only isolated particles contribute to the capacitance, when the amount of graphite reaches the percolation threshold the particle can be discharged and the capacitance decreases again, as can be observed in Figure 9, where the change in capacitance is presented for two selected frequencies (50 Hz and 1 kHz). The tendency of electrical capacitance to vary with frequency was similar to that of the electrical resistance. The capacitance attained a high value at a low frequency and decreased almost exponentially with an increase in frequency. The decrease in capacitance is mainly attributed to the mismatch of the interfacial polarization of composites to the external electric field at higher frequencies [42,43]. However, when the amount of graphite exceeded the percolation threshold, the influence of the frequency diminished and the capacitance of mixtures G20, G25 and G30 was even independent of the frequency. Based on the two independent relations concerning the effect of graphite content on electrical resistance and capacitance it was assumed that the percolation threshold was reached with approximately 12 wt% of graphite powder.

**Figure 10:**
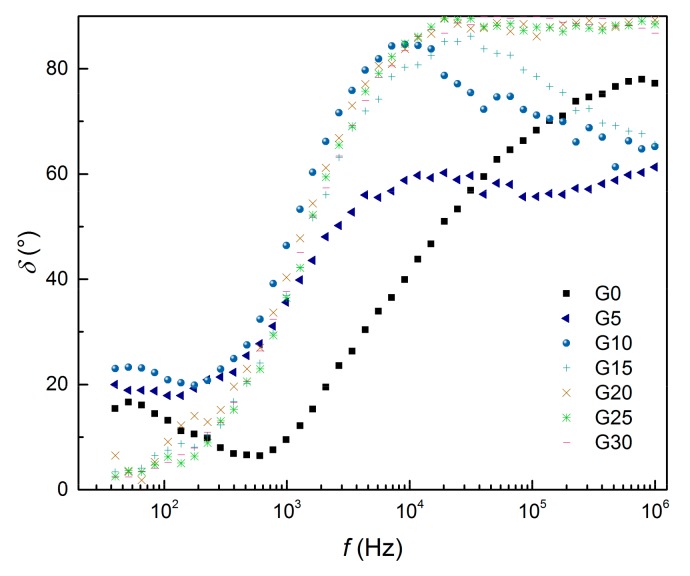
Loss angle of AAS composites with 0 wt%–30 wt% of graphite powder in the frequency range 40 Hz–1 MHz.

The loss angle *δ* determined for the complex impedance of AAS composites is presented in Figure 10, and for better distinguishability of the differences, only curves for selected mixtures (with graphite content increasing in 5% steps) were depicted. Low *δ* indicates that resistivity predominates, whereas at high *δ* the capacitance has the main controlling influence on the impedance. The loss angle generally increased with AC frequency, meaning that the imaginary part of the electrical impedance exceeds the real one at elevated frequencies. The local minimum was observed in the curve of the reference G0 mixture and its frequency directly corresponds to the peak in the resistivity curve. This indicates that in the range of 0.1–1 kHz the resistance strongly predominates over the capacitive reactance in the pure AAS mortar. When the graphite content was above the percolation threshold, at low frequencies, the impedance was controlled by the resistance component, but at frequencies above 10 kHz, the capacitive component predominated.

### 3.3. Self-Sensing Properties in Compression

The self-sensing properties of building materials have their origin in the alteration of the conductive pathways inside composite materials, so the changes in resistivity, expressed as the fractional change in resistivity (FCR), are able to measure the sensing behaviour. The main advantage of the FCR parameter is that it is independent from loading conditions, and can be calculated as follows:

(4) $$FCR(\%)=\frac{R-R_{0}}{R_{0}}\cdot 100,$$

where *R* is the electrical resistance and *R*_0_ is the initial electrical resistance. Self-sensing properties in compression were measured using both the DC and AC method. A total of 20 loading cycles were accomplished in the case of DC measurement and 10 cycles in the case of AC measurement in order to collect enough electrical resistance data. The loading range fits into the elastic part of the loading curve and hence should not cause any irreversible changes in the microstructure. For the assessment of self-sensing properties only the reference mixture G0 and two mixtures with different amounts of graphite were chosen. Since having a conductive filler concentration below the percolation threshold is beneficial for the sensing sensitivity under compression [1], a mixture with low graphite content (G3) and the mixture with graphite content almost reaching the percolation threshold (G10) were used in order to determine the effect of different filler content on the self-sensing properties.

**Figure 11:**
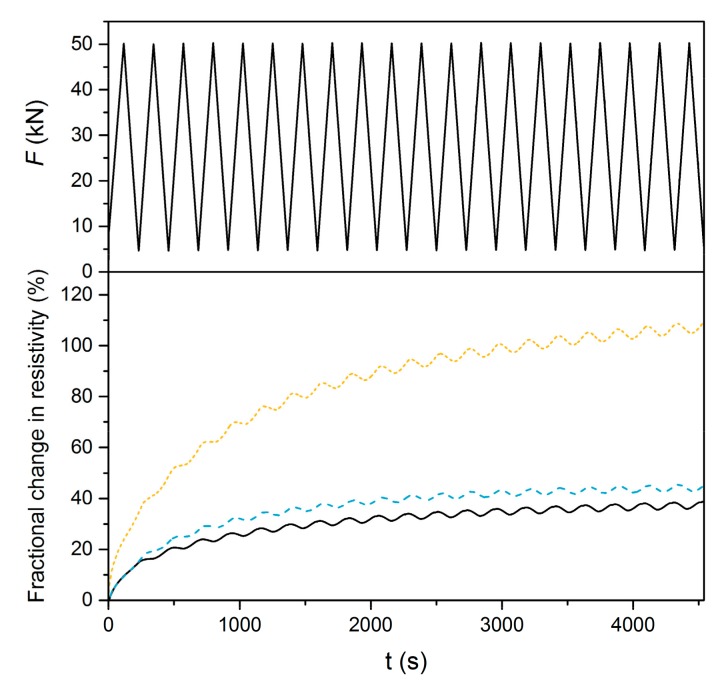
Compression sensitivity of composites G0, G3 and G10 under DC measurement.

Figure 11 shows the fractional change in resistivity during the first 20 cycles measured using the DC setup. It is obvious that all three curves exhibit a rising trend, which is most significant for the G10 mixture and is predominantly caused by the polarization effect. However, if we do not consider polarization, small variations in the FCR curves can be observed. As the compressive stress increased, the electrical resistance decreased, and vice versa, which is in accordance with studies reported for conductive cement-based composites [44,45,46,47]. When the compressive stress is applied, the resistance drops due to the closure of microcracks and defects in the matrix; during unloading, the defects are aggravated and the resistance increases again. The average difference between the local minimum and maximum for all three curves is 2.7%. This implies that the self-sensing properties of AAS are not sensitive to graphite content if the DC measurement is applied.

**Figure 12:**
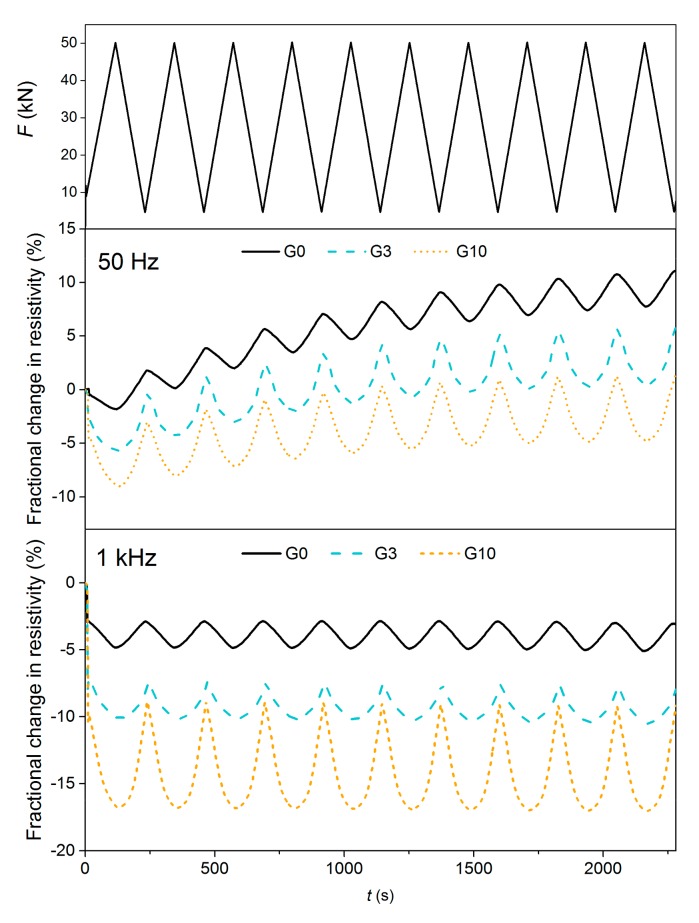
Compression sensitivity of composites G0, G3 and G10 under AC measurement at two different frequencies (50 Hz and 1 kHz).

The self-sensing properties under compression were also tested using AC measurement at two different frequencies, 50 Hz and 1 kHz (Figure 12). Compression sensitivity tested at 50 Hz still showed some polarization effect, although it was not as significant as with the DC measurement. The average difference between the local minimum and maximum increased for the G3 and G10 mixtures to 5.2% and 6.0%, respectively. Finally, no polarization occurred when the frequency of 1 kHz was applied. At this frequency, mixture G10 showed the highest sensing sensitivity, with an FCR interval of 8.6%. However, graphite filler had the adverse effect of causing aggravated sensitivity near the maximum loading force, which is represented by the U-shape of the FCR curve within one loading cycle. This is most significant with the G10 mixture for which the graphite content almost reached the percolation threshold. The abnormal decrease and non-linearity of FCR at the beginning of compressive loading between 0 and 0.5 MPa is attributed to the change in contact resistance, which is common for the two-probe method [48]. However, this phenomenon does not affect the ability to detect changes in resistance when the two-probe method is used [49,50].

**Figure 13:**
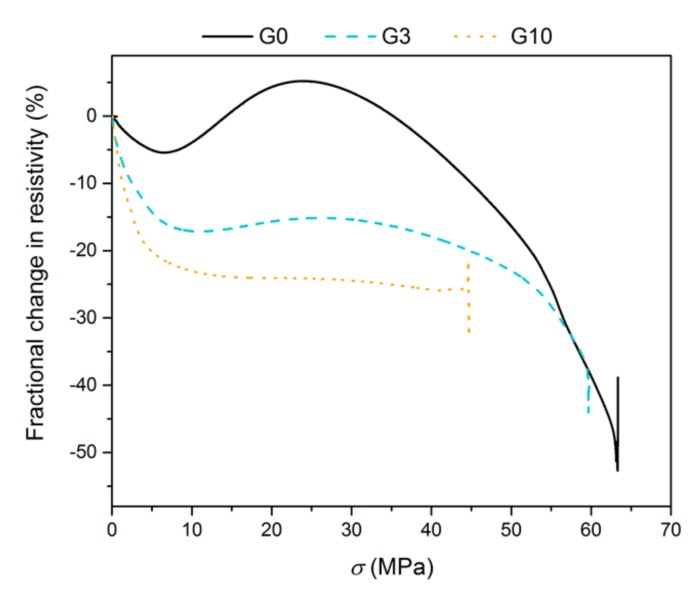
Fractional change in resistivity vs. compressive stress of composites G0, G3 and G10 measured at 1 kHz until the complete destruction of the specimens.

The measurement of self-sensing properties was also performed during monotonous compressive loading until failure (Figure 13). In order to avoid any polarization effect, the measurements were performed at 1 kHz. The curve recorded for the reference G0 mixture exhibited quite an extraordinary shape with a local maximum of FCR during loading. During the elastic part of loading the resistance linearly decreased, but then it increased again and reached the local maximum at 24 MPa. Thereafter, it gradually decreased again until failure. In the range of 14–35 MPa, positive values of FCR were achieved, and hence the resistance values even exceeded those of the reference composite. At the failure point the FCR increased abruptly, which was caused by the interruption of conductive pathways. Such behaviour has been previously reported for carbon nanotube reinforced cement composites [46]. It was also described in the authors’ previous work, where it was attributed to the formation of some preliminary defects which cause the opening of microcracks and the partial interruption of conductive pathways [48]. With the addition of graphite particles, the shape of the curve changed and the local maximum diminished. The G3 and G10 mixtures showed high sensitivity merely for very low compressive stress in the range of 0–6 MPa, and with higher stress the change in FCR was not significant. In particular, when the conductive admixture content almost reached the percolation threshold (G10 mixture), the sensitivity of the composite at a compressive stress of above 10 MPa disappeared. It is assumed that at a certain stress level the conductive particles become so close to each other that the system reaches maximum conductivity. The response of resistivity to the applied compressive stress is linear only up to 6 MPa and it is practically independent from the amount of graphite filler. This behaviour is associated with the elastic response of the composites to loading. Similar behaviour has already been reported by Han et al. [51] for a cement composite with nickel powder.

### 3.4. Microstructure

**Figure 14:**
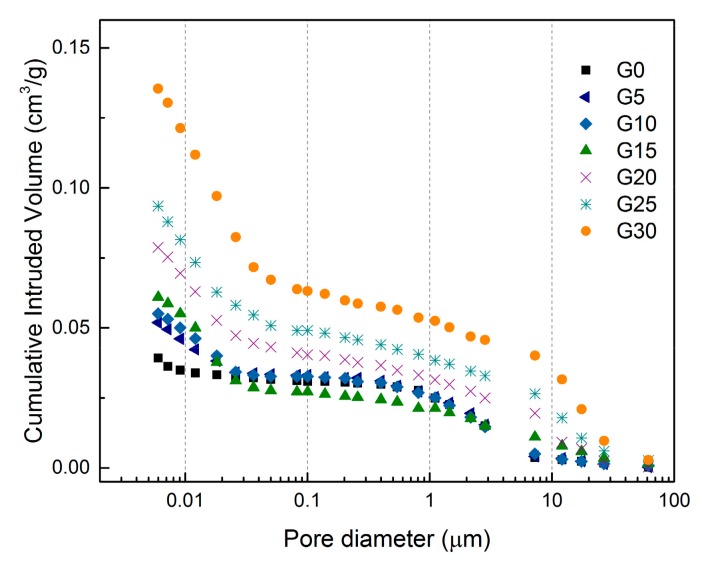
Differences in the pore distribution of selected mixtures of AAS mortars with graphite powder.

**Figure 15:**
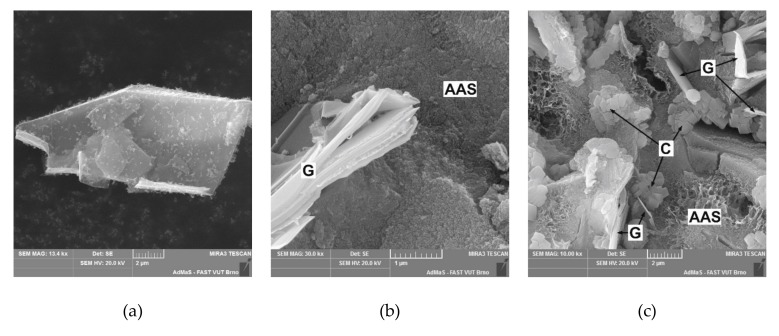
Details of microstructural features: (**a**) Shape of single graphite particle; (**b**) lamellar structure of graphite particle in the AAS matrix; (**c**) solid foam in the structure of AAS composite with 30 wt% of graphite. (AAS—alkali-activated slag matrix; G—graphite; C—calcium carbonate).

The microstructure of the AAS composites with conductive filler was analysed by means of scanning electron microscopy and mercury intrusion porosimetry. Pore distribution curves showing cumulative intruded volume versus pore diameter are depicted in Figure 14. The data show that the majority of the pore volume in the reference AAS mortar lies in the pore diameter range of 1–10 µm. After the addition of up to 10 wt% of graphite powder, only a small increase in pore volume associated with gel pores below 20 nm in diameter was observed. Greater amounts of graphite filler caused a remarkable increase in the volume of gel pores, as well as pores which are larger than 7 µm. This led to an overall increase in porosity, which was reflected in a drop in bulk density. The increase in volume, especially of large pores, can be also associated with the strength deterioration of the mixtures with the graphite content more than 10 wt%. The increase in volume of gel pores is probably associated with partial delamination of graphite platelets as can be observed in Figure 15 and partially it is influenced by the high amount of the organic additives which aggravate the formation of denser hydration products.

**Figure 16:**
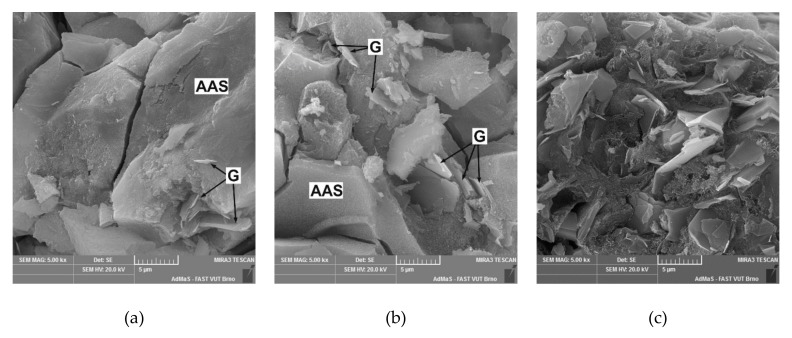
Microstructure of AAS composites with graphite filler—(**a**) 5 wt% graphite; (**b**) 10 wt% graphite; (**c**) 30 wt% graphite (G—graphite particles).

The morphology of the fracture surface of the AAS composites with conductive filler is presented in Figure 16. The figure shows the distribution of graphite particles in the AAS matrix. The microcracks located in the AAS gel are caused by drying shrinkage and were formed mainly during the preparation of the sample for the SEM imaging. The graphite particles are clearly visible as thin plates with a lamellar structure (Figure 15a,b). Since these particles are relatively sparsely dispersed in the AAS matrix containing just 5% graphite, only a few particles can be seen and the typical amorphous structure of alkali-activated slag predominates in the micrograph (Figure 16a). As the amount of graphite increases, the particles get much closer to each other, which is reflected in the enhancement of the specific conductivity. When 30 wt% of graphite is added, the particles occupy about 50% of the volume of the matrix and are in close contact with each other (Figure 16c). Such packing enables the AAS matrix to exhibit contact conductivity, and the limiting factor becomes the internal resistivity of the graphite. The increase in visible pores confirms the results obtained from the porosimetry measurements and can be attributed to the aggravated packing of plate-like particles on the one hand and the entraining of air bubbles due to the application of the Triton X-100 dispersant on the other. Although an air detraining admixture was added, some solid foam areas can be found in the micrographs at higher magnification (Figure 15c). The presented orthorhombic crystals can be attributed to calcium carbonate that is formed due to atmospheric carbonation of AAS matrix. This effect is most significant with G30 mixture which exhibits the highest porosity.

## 4. Conclusions

An investigation of the electrical and self-sensing properties of alkali-activated slag composite with graphite powder as a conductive filler is presented in this paper and the following conclusions have been drawn from the experimental results:

- The addition of graphite to AAS mortars leads to the deterioration of compressive strength, which can be attributed mainly to the resultant increased demand for mixing water and the consequent increased porosity of the AAS composite. The flexural strength is not affected unless more than 10 wt% of graphite is added, but then it significantly drops due to the weak bond between the graphite and the AAS matrix, and due to the lamellar structure of graphite particles.
- A considerable increase in the porosity of AAS composite determined by mercury intrusion porosimetry was achieved after the addition of graphite powder, especially when more than 10 wt% of graphite was present. This effect can be attributed mainly to the higher amount of water required in order to achieve the same workability as for the reference mixture, and also to the formation of solid foam due to the application of surfactant used for the better dispersion of hydrophobic graphite particles.
- The electrical resistivity of alkali-activated slag composite decreases as the amount of graphite powder and the frequency of the AC source increase; however, at frequencies over 100 kHz, the differences in the resistivities of all tested composites become quite negligible. The electrical capacitance rises when the amount of graphite powder increases until the percolation threshold is reached, whereupon it decreases again. It also generally decreases with AC frequency, but for the mixtures with very high graphite content it is frequency independent. Based on the influence of the conductive admixture on electrical resistivity and capacitance, the percolation threshold was assessed for the mixture containing approximately 12 wt% of graphite powder.
- Self-sensing properties were tested for a reference sample of pure AAS and two mixtures containing 3 wt% and 10 wt% of graphite powder in both the DC and the AC setup with two different frequencies (50 Hz and 1 kHz). Despite the large differences in resistivity, sensing properties were achieved for all three mixtures; however, the sensing sensitivity under repeated compressive loading depended on the frequency used. For the DC measurements, the sensitivity was independent of the amount of graphite admixture but the sensing properties were affected by strong polarization. In the case of the AC measurements at 1 kHz, the polarization was eliminated and the sensing sensitivity increased with the addition of graphite, being three times higher for the mixture with 10 wt% of graphite than for the reference mixture.
- The measurement of sensing properties under monotonous compression showed some extraordinary behaviour from the reference AAS mortar. After an initial drop in the fractional change in resistivity, a local maximum was observed, indicating some primary damage that occurred to the AAS matrix during compressive loading. After the addition of graphite, this maximum diminished. However, when the graphite content was near the percolation threshold (G10 mixture), the limiting conductivity was reached at 10 MPa and the sensitivity of the composite to compressive stress disappeared.
- The best sensing properties were achieved for the mixture with a graphite content almost reaching the percolation threshold, but the self-sensing properties were limited to very low compressive stress in the range of 0–6 MPa.

This study presented the effect of graphite admixture on the electrical and self-sensing properties of AAS composite, and the results can be used in the design of new types of smart materials. However, some aspects of the behaviour of AAS composites are yet to be explained, and hence further investigation and experimental work is needed in this field.

## Figures and Tables

**Table 1:** Chemical composition of granulated blast furnace slag (%).

SiO_2_	Al_2_O_3_	Fe_2_O_3_	CaO	MgO	K_2_O	Na_2_O	MnO	SO_3_
39.75	6.61	0.46	39.03	10.45	0.63	0.38	0.37	0.71

**Table 2:** Composition of the mixtures.

Mix	Slag	Water Glass	Sand	Graphite	Triton X-100	Lukosan S	Water
	(g)	(g)	(g)	(g)	(mL)	(mL)	(mL)
G0	450	90	1350	0	0	0	185
G1				4.5	30	5	150
G2				9	30	5	155
G3				13.5	30	5	160
G4				18	30	5	165
G5				22.5	60	10	135
G6				27	60	10	140
G7				31.5	90	15	110
G8				36	90	15	115
G9				40.5	120	20	85
G10				45	120	20	90
G15				67.5	180	30	75
G20				90	240	40	70
G25				112.5	300	40	70
G30				135	390	40	70
